# The Vital Statistics of Glasgow for 1843 and 1844

**Published:** 1846-07

**Authors:** 


### Art. XX.

*The Vital Statistics of Glasgow for* 1843 *and* 1844. Drawn up, &c. &c., by Alexander Watt, ll.d., City Statist.—*Glasgow,* 1846. pp. 148.

We noticed on a former occasion the valuable series of contributions to Vital Statistics, which emanated from the diligent pen of Dr. Watts, and of which the present is a constituent portion. It is not possible to notice here the sixty tables which Dr. Watt presents to the public in his closely-packed pamphlet; we must, therefore, refer the statist to the publication itself for details. Amongst the most interesting of these tables is a series exhibiting the fact, that there are specific laws which regulate the amount of deaths at different ages by the several diseases. These tables, Dr. Watt thinks, seem clearly to prove that, *cæteris paribus,* the mortality at different ages is uniformly in certain proportions to the amount of deaths by each disease respectively. To ascertain the precise effects of these laws, and the causes that may be brought into operation to produce a variation in their results, is, as Dr. Watt observes, of much importance, both for forwarding the science of vital statistics, and in the acquirement of a proper knowledge of the best modes of medical treatment, as well as for the advantages they must afford in coming to a correct estimate of the relative social conditions of the people of various localities. Illustrations in a tabular form of the operation of these laws are presented by Dr. Watt, derived from the Annual Reports of the Registrar-General. One of these tables shows, that although there was nearly double the amount of deaths by measles during the year 1840, in twenty-four town districts selected for comparison with the metropolis, than there was in the latter during the year 1842, the proportions at the same age are remarkably approximative. The difference under one year is 3·39 per cent.; this is the greatest, and it gradually becomes less at the higher ages. Under three years the difference is only 0·82 per cent.; under five years it is 0·58 per cent.; and under and above twenty years the difference is only 0·10 per cent. Scarlatina presents a similar proportionate mortality.

The causes of excessive mortality are considered by Dr. Watt, and in doing this he takes an opportunity to remark upon some critical observations we thought it expedient to make on some of his deductions in the report preceding this. It is gratifying to us to find that Dr. Watt appreciates our "spirit of fairness"—a spirit, we take leave to say, by no means foreign to the pages of this Journal—and that he acknowledges our competency to deal with the subject. On a careful perusal of Dr. Watt's statements, we do not feel it necessary to withdraw or modify the *juste milieu* opinions we then expressed. We stated it as our opinion

(No. XXXVI, p. 512) that much of the controversy respecting the causal relations of poverty to fever has originated partly in an indeterminate use of words, partly in misconceptions. We do not deny that the want of sufficient nutriment will render a person more liable to be attacked by fever, and will render the attack more severe, and more probably fatal. That is a point in pathology on which we could hardly imagine there were two opinions; and we hold also that the want of proper ventilation of the dwellings of the poor (so lamentably manifest) will increase the liability to, and add to, the mortality from fever. On this point we do not apprehend a dissentient voice. We are, however, most decidedly of opinion that the miasm arising from the extended cesspools of our large towns, termed sewers, and from open drains, when heat favours the decomposition of the animal and vegetable remains which they contain, will have two principal effects. Firstly, it will give increased virulency to any epidemic whatsoever that may be prevalent, whether it be measles, cholera, or typhus fever; and, secondly, it may so modify some of the ordinary epidemics that a new form or species of epidemic may arise. Thus a malignant remittent may be changed into a species of plague, as in some nautical epidemics, whether this modification occurs directly—simply by the reception of the miasm—or whether it be a new ferment generated by the miasm in a person whose blood is already contaminated by breathing the miasmatic atmosphere, may admit of question. The result of our investigations is, that it may and does occur in both ways. From some incidental remarks made by Dr. Watt, we should think he is not "well up" in the pathology of epidemical diseases, and that his sources of information are not such as to afford him large and comprehensive views on the subject.

The citizens of Glasgow must, however, feel much indebted to their city statist; and it is to be hoped that they will co-operate with the citizens of other large cities and towns of Scotland in a demand for an extension of the English system of registration to their own country. At present much of the labour expended by Dr. Watt on his tables is rendered effete by the want of the statistics of births, and of more exact and comprehensive nosology in the registration of the causes of death. When we find such entries as "bowel hives," "bloody flux," "shortness of breath," &c., among the causes of death, we cannot but attach much uncertainty to the pathological statistics derived from so questionable a source.

---

### ART. XXI.

*Essai sur les Tumeurs Solides Intra-Thoraciques.* Par J. M. H. GINTRAC. Thèses de Paris, Janv. 1845.

*Essay on Solid Intra-Thoracic Tumours.* By J. M. H. GINTRAC, &c. &c. 4to, pp. 67.

